# A Single-Image Noise Estimation Algorithm Based on Pixel-Level Low-Rank Low-Texture Patch and Principal Component Analysis

**DOI:** 10.3390/s22228899

**Published:** 2022-11-17

**Authors:** Yong Li, Chenguang Liu, Xiaoyu You, Jian Liu

**Affiliations:** 1 Research Center of Advanced Microscopy and Instrumentation, Harbin Institute of Technology, Harbin 150001, China; 2 Research Center of Basic Space Science, Harbin Institute of Technology, Harbin 150001, China

**Keywords:** image processing, image denoising, noise level, white Gaussian noise, clustering algorithm

## Abstract

Noise level is an important parameter for image denoising in many image-processing applications. We propose a noise estimation algorithm based on pixel-level low-rank, low-texture subblocks and principal component analysis for white Gaussian noise. First, an adaptive clustering algorithm, based on a dichotomy merge, adaptive pixel-level low-rank matrix construction method and a gradient covariance low-texture subblock selection method, is proposed to construct a pixel-level low-rank, low-texture subblock matrix. The adaptive clustering algorithm can improve the low-rank property of the constructed matrix and reduce the content of the image information in the eigenvalues of the matrix. Then, an eigenvalue selection method is proposed to eliminate matrix eigenvalues representing the image to avoid an inaccurate estimation of the noise level caused by using the minimum eigenvalue. The experimental results show that, compared with existing state-of-the-art methods, our proposed algorithm has, in most cases, the highest accuracy and robustness of noise level estimation for various scenarios with different noise levels, especially when the noise is high.

## 1. Introduction

Noise level is a crucial parameter in many imaging applications, such as super-resolution [1,2,3,4], image segmentation [5,6], and other applications [7,8,9,10]. Particularly in the field of image denoising, the standard deviation of noise must be precisely known to obtain a good denoising effect. However, the noise level is unknown in practice. Therefore, it is necessary to estimate the noise level before denoising, and the accuracy of the estimation directly affects the performance of the denoising algorithm. In recent decades, many noise estimation algorithms have been proposed.

The model of a noisy image is generally expressed as Y=X+N, where *Y* ∈ *R^a^*^×*b*^ represents the image polluted by noise, *X* ∈ *R^a^*^×*b*^ represents the image without noise, and *N* ∈ *R^a^*^×*b*^ represents the additive white Gaussian noise with a mean of 0 and standard deviation σ. Gaussian noise is commonly used as an object because (1) Gaussian noise is the most commonly encountered type of noise in the imaging process, and (2) a substantial amount of noise can be transformed into Gaussian noise through certain methods [11,12,13]. The goal of noise estimation is to determine the standard deviation of the noise. Currently, noise estimation methods are generally divided into three categories: transform domain- [14,15,16,17,18,19,20,21,22,23,24,25,26], filter- [27,28,29,30,31], and patch-based methods [32,33,34,35,36,37,38,39,40].

Transform domain-based methods transform the image into other domains to separate the image information from the noise information to a certain extent, and then use some coefficients, such as eigenvalues, kurtosis values, and wavelet coefficients, to estimate the noise. Donoho et al. [14] converted the image to the wavelet domain, assuming that the wavelet coefficients in the first diagonal sub-band are dominated by noise, and computed the noise level as the scaled median of these absolute coefficients. Rahmat et al. [15] directly calculated the standard deviation of the noise using the median absolute deviation (MAD) of the wavelet coefficient of the first sub-band. This method may cause the standard deviation of the noise to be overestimated because the first sub-band often contains image information. Moreover, this method only verifies the case when the noise level, σ, is lower than 25. To further reduce the negative impact resulting from strong image structures, Li et al. [16] suggested computing the wavelet coefficient of the Canny edge map from a noisy image, which is used to steer the exclusion of wavelet coefficients dominated by image signals. The authors verified that the proposed method was more accurate than the MAD method in the case of low noise. Similarly, Pimpalkhute et al. [17] used a hybrid discrete wavelet transform (DWT) and an edge information removal-based algorithm to estimate the level of Gaussian noise. In this method, the corresponding image edge information in the wavelet coefficients was first removed, and then the standard deviation of the noise was solved using the root mean square of the wavelet coefficients. Finally, polynomial regression was used to further improve the accuracy of estimation, but the performance of the algorithm was affected by the polynomial regression coefficients. Liu et al. [18] used singular value decomposition to obtain the singular value matrix of the noisy image, and then constructed a linear relation formula between the singular value and the standard deviation of the noise. By solving this formula, the standard deviation of the noise was calculated, which was suitable for σ ≤ 50. Ponomarenko et al. [19] divided an image into 8 × 8 blocks, applied a discrete cosine transform (DCT) to these blocks, and filtered the DCT coefficients. Finally, the MAD was used to calculate the standard deviation of the noise for the screened coefficient directly. This method can obtain relatively high accuracy when the noise is low (σ ≤ 15). Zoran et al. [20], Wu et al. [21], and Li et al. [22] linked noise variance with kurtosis values. Finally, noise level estimation was cast into a nonlinear optimization problem. However, these methods can only estimate a noise level of σ ≤ 50.

In filter-based methods, a noisy image is high-pass filtered to suppress image information, and the filtered image is considered to contain only noise information. Immerkær et al. [27] used high-pass filters to filter out image information from noisy images and then directly averaged the obtained noisy images to calculate the standard deviation of noise. However, the filtered image must contain a large amount of image information; therefore, the accuracy of the standard deviation of the noise obtained by this algorithm is not high. To improve the performance of the filter-based noise estimation algorithm, several improved algorithms [28,29] have been proposed. Researchers used a Laplacian to suppress the structural information of the image and exclude the edge information by using an edge detection operator. However, because the filtered image always contains image information, particularly when the image is complex, the noise level is often overestimated.

Patch-based methods play an important role in noise estimations. In general, patch-based methods first divide images into blocks, then select patches to compose low-rank sub-image matrices, and finally estimate the standard deviation of noise. Jiang et al. [40] selected flat blocks iteratively, then performed eigenvalue decomposition on a low-rank matrix composed of flat blocks, and finally used the minimum eigenvalue as the standard deviation of noise. Liu et al. [33] constructed a low-rank subblock matrix by extracting the low-texture subblocks of an image and used the minimum eigenvalue as the estimated value of the standard deviation of noise. Fang et al. [38] proved that the algorithm proposed by Liu et al. [33] often underestimated noise, whereas the method proposed by Jiang et al. [40] often overestimated noise. Fang et al. used a formula to combine the minimum eigenvalues obtained by the two methods as the final estimation value of the noise. The method proposed by Khmag et al. [37], which is similar to that proposed by Liu et al. [33], also uses the minimum eigenvalue of the low-rank subblock matrix as the estimated value of noise. The method proposed by Chen et al. [39] does not use the minimum eigenvalue to calculate the standard deviation of noise but uses the average value of some eigenvalues as the estimation value of noise. Thus, the results can be more accurate, but they are used in color images and may fail in gray images [40]. Although all of these patch-based methods are used to extract homogeneous patches, flat patches, or low-texture patches, in many cases, there are no or few flat or low-texture patches in actual images, which seems to lead to a large deviation in the calculation results. However, this patch-based and principal component analysis (PCA)-based method only requires that the sub-image blocks in the low-rank subblock matrix be similar image blocks, without requiring the subblock images to be homogeneous regions, flat blocks, or low-texture blocks. The methods above can achieve relatively accurate results with a small amount of noise, but this is not necessarily the case with a large amount of noise.

Therefore, the patch-based noise estimation algorithm has two main problems: (1) the estimated noise value is overestimated or underestimated, and (2) the algorithm achieves a good effect at low and medium noise levels, but has poor effect or no conclusion at high noise levels. The causes of these two problems are that (1) the constructed low-rank or low-texture matrix still contains a lot of image information, and (2) the estimation algorithm of noise is not accurate. We propose a new patch-based noise estimation method that has high accuracy and robustness not only at low noise levels but also at high noise levels. First, instead of directly selecting flat blocks or low-texture blocks in the noisy image, we first use an adaptive clustering algorithm based on dichotomy merge for the noisy image to construct a low-rank sub-image block matrix. On this basis, we use the improved pixel-level matrix construction method derived from Hou [40] to build the pixel-level low-rank sub-image block matrix adaptively. Then, we use the gradient covariance method to select the low-texture block matrix on the basis of the pixel-level low-rank image block matrix. In this manner, the low-rank property of the constructed low-rank sub-image blocks can be further improved, and the eigenvalues of the matrix contain less image information. Second, PCA technology is used to solve the standard deviation of the noise. The difference is that we eliminate the eigenvalues representing the image information through the proposed eigenvalue selection method and use the average value of the remaining eigenvalues to calculate the standard deviation of noise instead of using the minimum eigenvalue. Using the average value of eigenvalue to estimate the standard deviation of noise can effectively reduce the error caused by a single eigenvalue. At the same time, the eigenvalue selection method can effectively eliminate the eigenvalue representing the image information, which makes the noise estimation more accurate. Even in the case of high noise, our algorithm can still achieve satisfactory results. In addition, when the standard deviation of the noise is calculated using the block method, a certain number of pixels is required, because if the number of pixels is too small, the estimation result will have a large deviation. According to the standard deviation and error tolerance of noise, we propose an adaptive method to calculate the number of pixels required to calculate the standard deviation of the noise.

## 2. Method

### 2.1. Formulation

Suppose a Gaussian noise matrix *N* with mean 0 and standard deviation σ. Each element in the matrix follows a Gaussian distribution and is independent of each other, that is,

(1) $$N=\left[\begin{array}{ccc} n_{11} & \ldots & n_{1b}\\ \vdots & \ddots & \vdots \\ n_{a1} & \ldots & n_{ab} \end{array}\right],n_{i}{}_{j}\in N(o,\sigma^{2}),i=1\ldots a,j=1\ldots b$$

The value of each element in the noise matrix is known, but the standard deviation σ representing the noise level is unknown. Suppose a set of variables *A* = {*a*_1_, *a*_2_… *a*_n_}, where each variable is independent of the others and follows the standard normal distribution, that is, *a*_i_ *~ N*(0, 1). Therefore, according to the definition of the chi-square distribution, *A* satisfies

(2) $$\sum_{i=1}^{n}a_{i}^{2}∼\chi^{2}(n)$$

Then, we have

$$\frac{1}{ab}\sum_{i=1}^{a}\sum_{j=1}^{b}n_{ij}^{2}=\frac{1}{ab}\langle N,N\rangle =\frac{1}{ab}\sum_{i=1}^{\min(a,b)}\sigma_{i}^{2}\overset{}{∼}N(\sigma^{2},\frac{2\sigma^{4}}{ab})$$

where $N=U\Lambda V,\Lambda =diag(\sigma_{1},\sigma_{2}\ldots \sigma_{\min(a,b)})$, and <> denotes the inner product. According to Equation (5), the standard deviation of the noise can be expressed as

(3) $$\sigma =\sqrt{\frac{1}{ab}\sum_{i=1}^{\min(a,b)}\sigma_{i}^{2}}$$

Assume that a matrix *X* ∈ *R^a^*^×*b*^ is polluted by a Gaussian noise matrix *N* ∈ *R^a^*^×*b*^ with zero mean and standard deviation σ; therefore, the noise-polluted matrix *Y* ∈ *R^a^*^×*b*^ can be represented as

(4) Y=X+N.

Here, the matrix *Y* polluted by noise is measured, whereas the noise matrix *N* is unknown. Eigenvalues must be used to calculate the standard deviation of the noise. There are two main ways to calculate the eigenvalues, singular value decomposition (SVD) and PCA, which are consistent. According to the definition of the eigenvalues, the eigenvalue decomposition of a matrix projects the matrix onto another basis, and the corresponding coefficients of this basis are the eigenvalues. Based on PCA, we can define the direction of the basis using unit vector *u_i_*. The signal and noise are uncorrelated. In addition, Gaussian noise is independent and random; therefore, its corresponding coefficients in each principal component direction should be approximately equal in theory. Then, the projection of matrix in this direction is expressed as

(5) $$\lambda_{i}=W(u_{i}^{\prime }Y)=W(u_{i}^{\prime }X)+\sigma^{2}$$

where *λ_i_* is the *i*th eigenvalue, *u_i_* represents the eigenvalue value corresponding to the *i*th eigenvalue, ${\left(\right)}^{\prime }$ represents the transpose operator, and *W* represents the projection operator. To obtain the standard deviation of the noise, the direction corresponding to the minimum eigenvalue should first be calculated. Thus,

(6) $$u_{\min}=\arg\underset{u_{i}}{\min}W(u_{i}^{\prime }Y)=\arg\underset{u_{i}}{\min}W(u_{i}^{\prime }X).$$

According to the definition of PCA, this direction is exactly the direction corresponding to the smallest eigenvalue of the matrix covariance; that is,

(7) $$\lambda_{\min}(\sum_{Y})=\lambda_{\min}(\sum_{X})+\sigma^{2}$$

where $\sum_{Y}=YY^{\prime }$ represents the covariance matrix of the matrix containing noise and $\sum_{X}$ represents the covariance matrix of the noiseless matrix. According to the above equation, the minimum eigenvalue can be easily calculated, and the noise level can then be estimated. However, because the noiseless matrix cannot be known in practice, its minimum eigenvalue cannot be determined; therefore, the above problem is actually an ill-posted problem. Nonetheless, if the noiseless matrix is of low rank, the minimum eigenvalue of the noiseless matrix is approximately zero. At this point, we can approximate the standard deviation of the noise as follows:

(8) $${\overset{⌢}{\sigma }}^{2}=\lambda_{\min}(\sum_{Y}),$$

where $\overset{⌢}{\sigma }$ represents the estimated standard deviation of noise. All the existing methods of noise level estimation based on eigenvalues are derived from this.

To improve the accuracy of the noise standard deviation estimation, it is necessary to obtain a low-rank subblock composed of image blocks. The image subblocks are required to be not only low-rank, but also low-texture for the more available data.

**Figure 1:**
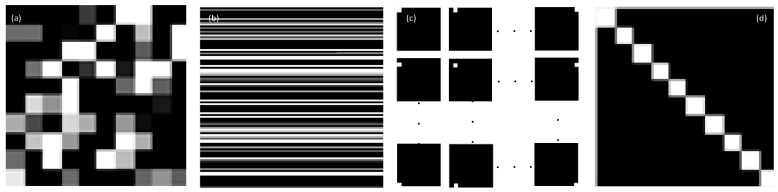
Difference between low rank and low texture. (**a**) high-texture image block, (**b**) low-rank high-texture matrix, (**c**) low-texture image blocks, (**d**) full-rank low-texture matrix.

Notably, there is no relationship between low-rank and low-texture subblocks; the sub-images in low-rank subblocks are not necessarily low-texture, and the matrix composed of low-texture image blocks is not necessarily low-rank, as shown in Figure 1. It can be observed that as a sub-image block, Figure 1a has obvious texture information, which clearly belongs to a high-texture image block. The sub-image matrix shown in Figure 1b belongs to a high-texture matrix, but it is a low-rank matrix because the image similarity is very high. Each sub-image block in Figure 1c is obviously a low-texture image block because each image block has only one position where the value is not zero. After vectorization, the sub-image matrix constructed by stacking them is obviously a low-texture matrix, but it has full rank, as shown in Figure 1d. Therefore, low rank and low texture are not the same concept.

### 2.2. Proposed Method

**Figure 2:**
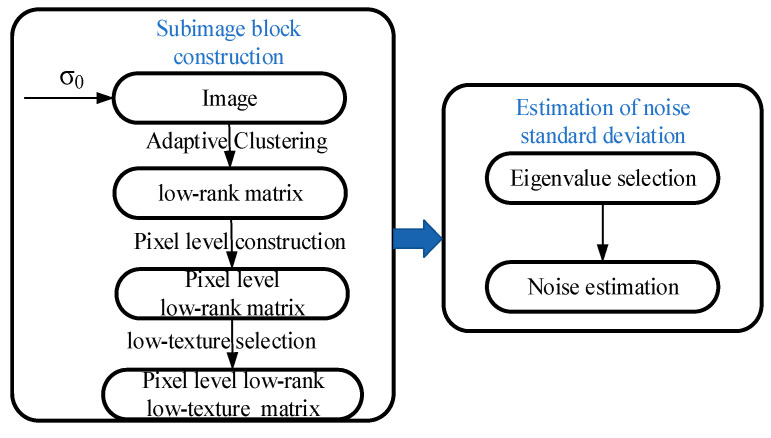
The flowchart of our algorithm.

The flow chart of our proposed algorithm is shown in Figure 2, which is mainly divided into two steps. One step is the construction of pixel-level low-rank, low-texture sub-image block matrix, and the other step is the estimation of noise. See Section 2.2 and Section 2.3 for details of the algorithm.

#### 2.2.1. An Adaptive Clustering Method Based on Dichotomy Merge

Our algorithm must extract similar sub-images of low texture from the image to form a low-texture, low-rank sub-image matrix. According to the non-local self-similarity of images, images contain a large number of similar structures. There are generally two methods for selecting similar structures: sub-image matching [27,28] and clustering [41,42,43]. Clustering was used in this study.

Cluster analysis is a multivariate statistical method that classifies research objects based on certain characteristics. It has been successfully applied to economics, medicine, meteorology, and other fields. It does not consider the causality between characteristics and variables. The clustering results show that the individual differences between the categories are large, whereas those within the same category are relatively small. The most commonly used clustering method is *K*-means clustering. First, *K* image blocks are randomly selected from the image as the initial centroid, the distances between other image blocks and these centroids are calculated, and the image blocks are classified according to different distances. The mean of the image blocks within the class is calculated as the new centroid until the termination condition is satisfied. The *K*-means algorithm is widely used because it is simple and has a relatively good clustering effect. In general, a clustering algorithm should be able to distinguish different features in the image and group the same features together. However, the traditional *K*-means clustering algorithm cannot meet this requirement owing to its two defects. First, the initial centroids are completely random. In extreme cases, these centroids may all be in a class, leading to incorrect clustering results. Second, the optimal number of clusters cannot be determined because it is a fixed value.

Substantial research has been conducted to address these two shortcomings. Regarding the first defect, the best-known improved algorithm is the *K*-means ++ clustering algorithm, which can be summarized as selecting K cluster centers step by step and calculating the distances between the sample points in a non-class and the existing cluster centers. The greater the distance, the more likely it is to be selected as the next cluster center. Regarding the second defect, the best-known clustering method is the one that adopts the idea of “divide and conquer”. This type of method generally divides the image into sufficient classes first, that is, over-clustering, and then reduces the number of clusters by merging.

Therefore, an adaptive clustering method based on dichotomy merge is proposed that can adaptively select the centroid and cluster number. Different features in images must have external manifestations, usually in the form of differences in the mean or variance. We use the product of the two as the selection condition of the initial centroids; the larger the difference between the product values, the lower the probability of belonging to the same feature. Therefore, the two sub-images with the largest differences are selected as the initial clustering centers. The K-means algorithm is then used to classify other sub-images into these two classes, but this inevitably leads to the clustering of different features into the same class. We then use the above method to cluster each of the two classes again and obtain four new classes. If the inter-class distance between the four classes is calculated to be less than a certain value, we consider them to belong to the same class and should merge them. The above process is continued until the termination condition is reached, which is generally set to no change in the number of classes or a certain number of iterations.

It can be observed that the number of clustering centers is determined by the inter-class distance threshold, which affects the clustering results. The main reason for incorrect clustering is noise; therefore, this threshold should be positively correlated with noise. Suppose that there are two different classes, *Clas_A* ∈ *R^m^*^×*la*^ and *Clas_B* ∈ *R^m^*^×*lb*^, after image clustering, and the centers of these two classes are *y_A_* ∈ *R^m^*^×1^ and *y_B_* ∈ *R^m^*^×1^. The class center is the mean vector of each sub-image vector within the class, *y_A_* = *x_A_* + *n_A_*, *y_B_* = *x_B_* + *n_B_*, where x_A_ and x_B_ are the mean vectors of the sub-image block vector without noise within the class, ***n****_A_* =$\left[n_{A}^{1},n_{A}^{2},\ldots ,n_{A}^{m}\right]$ and ***n****_B_* = $\left[n_{B}^{1},n_{B}^{2},\ldots ,n_{B}^{m}\right]$ are the mean vectors of the noise vector within the class, $n_{A}^{i}=\frac{1}{la}\sum_{j=1}^{la}n_{j}^{i}$ ∈ *N*(0, *σ*^2^/*la*),$n_{B}^{i}=\frac{1}{lb}\sum_{j=1}^{lb}n_{j}^{i}$ ∈ *N*(0, *σ*^2^/*lb*), and $n_{j}^{i}$∈*N*(0, *σ*^2^); therefore, the distance between the two classes is

(9) $$D={\left\|y_{A}-y_{B}\right\|}_{F}^{}={\left\|x_{A}-x_{B}+(n_{A}-n_{B})\right\|}_{F}^{}.$$

Thus, the probability of merging the two classes should be *P*(*D* < *T*) = 1 − ε, and it is difficult to solve the above formula directly. As in [41], we assume an extreme case in which the two classes have the same features. In this case, we assume that *x_A_ = x_B_*, *lb* = 1, and *la*→∞. Equation (12) can be then derived as $D={\left\|n_{B}\right\|}_{F}$. *D*^2^ follows a chi-square distribution with *m* degrees of freedom. When *m* = 100 and *ε =* 9 × 10^−13^, we can obtain T = 30σ^2^. However, because we have made many limiting assumptions, the actual threshold should be smaller than the above threshold. In the experiment, the threshold parameter is set as 12*σ*^2^. When the square of the distance between two classes is less than the threshold parameter, they should be classified as one class.

In addition, in the actual experiment, with an increase in noise, the image features are gradually submerged. At this time, the number of classes divided by the clustering algorithm gradually decreases, and the number of elements in the classes gradually increases. This resulted in the inclusion of different features in the classes, and the low-rank property of the constructed low-rank sub-image block vector matrix is reduced. Therefore, as noise increases, the threshold parameter should be further reduced.

The task of our clustering is to find a low-rank subblock initially. The number of similar image subblocks in the low-rank subblock should be as large as possible because the low-texture subblocks should be screened on the basis of this low-rank subblock in the future. Thus, it needs to contain a certain number. The clustering algorithm is summarized in Algorithm 1.
**Algorithm 1:** Adaptive clustering algorithm based on dichotomy mergeInput: Noisy image *Y*, standard deviation of noise *σ,* size of image batch *c,* maximum number of iterations *K.*Output: Low-rank sub-image block group.Steps:1. The noise image is divided into blocks with the size of *c* × *c*, and the adjacent image blocks are only different by one row or one column;2. While the class center does not change or the number of iterations *k* = *K*For each class     1. The mean and variance of each image block in the class are calculated and combined as the centroid judgment factor;     2. The two image blocks with the largest difference in centroid judgment factor are selected as the two initial class centers;     3. The K-means algorithm is used for clustering, and the class center of each class is calculated;End for4. The distance between classes is calculated, and the merging threshold is used to determine whether the class needs merging;5. *k* = *k* + 1;End while

#### 2.2.2. Construction Method of Pixel-Level Low-Rank Image Subblock Matrix

In the existing methods for constructing a low-rank sub-image matrix, whether the method of block matching, clustering, or low-texture selection, they first vectorize the sub-image, then judge the similarity between the sub-image column vectors according to certain criteria, and then stack the similar sub-image column vectors. Each column of the sub-image matrix constructed by this method is considered to be similar, and its similarity is less than a certain threshold. The smaller the similarity is, the better the low rank of the constructed sub-image matrix. 

Hou [36] proposed the construction of a pixel-level low-rank matrix that screens the rows and columns. In other words, it is necessary to calculate the similarity between the rows of the low-rank subblock *Y_k_* ∈ *R^m^*^×*k*^. If any row $y_{k}^{i}$ ∈ *R*^1×*k*^ is selected from the low-rank subblock *Y_k_*, the formula for calculating the similarity between it and the *j*th row $y_{k}^{j}$ is as follows:

(10) $$d_{k}^{ij}=\sqrt{\frac{{\left\|y_{k}^{i}-y_{k}^{j}\right\|}_{F}^{2}}{k}}$$

In the method proposed by Hou [36], a fixed number of rows with the smallest value of the similarity measure function is selected to construct a new low-rank subblock. However, this is obviously not sufficiently accurate because different noise levels of different images will inevitably lead to different numbers of similar rows. We still consider an extreme case, assuming that the noiseless image is texture-free and that the noisy image contains only noise information. Thus,

(11) $$\sqrt{\frac{{\left\|y_{k}^{i}-y_{k}^{j}\right\|}_{F}^{2}}{k}}=\sqrt{2}\sigma$$

When the number of columns in the low-rank sub-image block matrix is sufficiently large and the corresponding noiseless sub-image block is completely flat, the threshold *τ* of the inter-row similarity of the low-rank sub-image matrix is $\tau =\sqrt{2}\sigma$. In practical applications, because the number of *k* is limited and the image itself contains texture information, we introduce a magnification factor η into *τ*:

(12) $$\tau =\eta \sqrt{2}\sigma .$$

It can be observed that the threshold is related to noise; therefore, it is more reasonable than the method of [36] to select a fixed number directly. When the value of the similarity measure function is not greater than the threshold, the row is considered to be sufficiently similar to the reference row and is called a similar row, and all the similar and reference rows are retained to construct a new low-rank subblock. The algorithm is summarized in Algorithm 2.
**Algorithm 2:** Construction method of pixel-level low-rank image subblock matrixInput: Initial low-rank image subblock matrix *Y_k_*, standard deviation of noise *σ,* minimum number of similar rows N, zoom factor *η*Output: Pixel-level low-rank image subblock matrixSteps:1. The row $y_{k}^{c}$ with the smallest variance in the initial low-rank image subblock matrix is calculated as the reference sub-image block vector;2. For all row vectors $y_{k}^{i}$ of the initial low-rank image subblock matrix     1. Calculate the similarity measure function: $d_{k}^{ci}=\sqrt{\frac{{\left\|y_{k}^{c}-y_{k}^{i}\right\|}_{F}^{2}}{k}}$;     2. Calculate the threshold: $\tau =\eta \sqrt{2}\sigma$;     3. If $d_{k}^{ci}\leq \tau$, the row is kept as a similar row; End for3. If the number of rows of the output pixel-level low-rank image sub-block matrix is less than N, increase the scaling factor *η* = *η* + 0.01 and go to step 2;4. Output the pixel-level low-rank image subblock matrix

#### 2.2.3. Low-Texture Subblock Selection Method Based on Gradient Covariance

The low-rank sub-image matrix *Y* = {*y*_1_, *y*_2_… *y_l_*} ∈ *R^m^*^×*l*^ with similar features was obtained using the above adaptive clustering algorithm. Each of these sub-images *y_i_* can be expressed as *y_i_* = *x*_i_ + *n*, where ***x***_i_ represents the noiseless sub-image block, and *n* represents the Gaussian noise matrix with zero mean and noise standard deviation *σ*. Therefore, it is also necessary to select the low-texture subblock in the low-rank sub-image matrix to construct a low-texture, low-rank sub-image matrix. To select low-texture subblocks from low-rank subblocks, it is necessary to analyze the texture structure information of noisy sub-images. The variance can better represent the texture intensity of images; therefore, many scholars use variance as an index to judge the strength of textures. Lee et al. [44] calculated the local variance of the sub-image and selected the smallest one as the low-texture sub-image. The method of using variance as a standard is simple and rapid. However, when the image noise is relatively high, the sub-images with rich texture tend to be mixed into the sub-image group with low texture, resulting in relatively high noise estimation results. Shin et al. [31] used an adaptive threshold to select subblocks with a low texture; however, the actual effect was unsatisfactory. Therefore, it is not accurate to use variance alone as an index to judge texture richness. Similarly, edge or corner information, when used as the evaluation index, is susceptible to noise. Zhu et al. [45] proposed that the structural information of an image can be measured by the gradient covariance matrix, and the result is more stable than the method using variance as the index. Therefore, Liu et al. [33] and Fang et al. [37] proposed noise estimation and denoising algorithms. Our low-texture sub-image selection method takes this approach.

First, we assume that noiseless image subblock ***x***_i_ is a flat subblock. The simple gradient operator is used to calculate the gradient of the low-rank subblock matrix.

(13) $$\begin{array}{l} G_{y_{i}}=[\begin{array}{cc} FL_{h}y_{i} & FL_{v}y_{i}] \end{array}=[\begin{array}{cc} FL_{h}(x_{i}+n) & FL_{v}(x_{i}+n)] \end{array}\\ =[\begin{array}{cc} FL_{h}n & FL_{v}n] \end{array} \end{array}$$

where *FL_h_* and *FL_v_* represent the horizontal- and vertical-derivative operators, respectively. The specific forms of these two derivative operators can be derived from simple filter construction [45]. In this paper, we take *FL_h_* and *FL_v_* as [−1/2, 0, 1/2] and [−1/2, 0, 1/2]. According to [33,46], the statistical characteristics of the texture intensity of the Gaussian noise approximately follow the gamma distribution:

(14) $$\xi ∼Gamma(\frac{m}{2},\frac{2}{m}\sigma^{2}tr(FL_{h}^{\prime }FL_{h}+FL_{v}^{\prime }FL_{v})),$$

where $\xi =tr(G_{y_{i}}^{\prime }G_{y_{i}})=n^{\prime }(FL_{h}^{\prime }FL_{h}+FL_{v}^{\prime }FL_{v})n$ represents the texture strength, *tr*() denotes the trace operator, and *Gamma*(*a*, *b*) represents the gamma distribution with shape parameter *a* and scale parameter *b.* The texture intensity *ξ* represents the structural information of the sub-image block. When it is less than a certain value, we consider it to be a low-texture subblock. The threshold *τ* is given as

(15) $$\tau =\sigma^{2}Gamma^{-1}(\delta ,\frac{m}{2},\frac{2}{m}tr(FL_{h}^{\prime }FL_{h}+FL_{v}^{\prime }FL_{v})),$$

where *Gamma*^−1^(*δ*, *a*, *b*) represents the inverse gamma distribution function with a significance level *δ*, shape parameter *α,* and scale parameter *b*. The meaning of this significance level *δ* is the probability that the texture strength is between 0 and *τ*. This significance level is close to 1. That is to say, when the texture strength of sub-image block is less than this threshold *τ*, the sub-image block is a low-texture sub-image block.

Therefore, the judgment criterion of a low-texture sub-image is

(16) ξ≤τ.

Because these low-texture sub-image blocks are selected from the low-rank sub-image blocks after clustering, the constructed sub-image matrix can be guaranteed to be a low-texture, low-rank matrix to a large extent.

#### 2.2.4. Eigenvalue Selection Method

After obtaining the pixel-level low-rank, low-texture sub-image block matrix $Y_{k}^{iq}$ ∈ *R^iq^*^×*k*^, the standard deviation of the noise is calculated according to Equation (3). However, the derivation of Equation (3) only considers the noise matrix. The noiseless subblocks in the constructed low-rank subblocks cannot be completely texture-free; therefore, the eigenvalues in the equation must also contain image information. Fortunately, the constructed pixel-level low-rank, low-texture sub-image block matrix is a type of low-rank sub-image block matrix; therefore, the image information mainly focuses on the larger eigenvalues, while the noise eigenvalues are much smaller and more stable. Therefore, as long as the larger eigenvalues representing the image information are removed, the remaining eigenvalues are used to calculate the standard deviation of noise. According to the literature [37] or the Grubbs criterion, when the eigenvalues of the matrix meet certain conditions, the eigenvalues of the matrix can be judged to have upper outliers through the following conditions:

(17) $$if\{\begin{array}{cc} mean(\sum )\geq median(\sum ) & t=1\\ mean(\sum )<median(\sum ) & t=0 \end{array},$$

where *mean*() represents the mean and *median*() represents the median. *∑* = {*σ*_1_... *σ_iq_*} denotes the singular value vector of the matrix, and *t* denotes the upper outlier identifier.

When *t* = 1, the largest eigenvalue is an outlier, and must be removed. The above judgment is executed iteratively until the condition *t* = 0 is satisfied. In this manner, *ip* eigenvalues satisfying the conditions can be obtained, and *ip* ≤ *iq*. However, the number of eigenvalues determined by this condition is sometimes excessively large. According to this observation, we find that when there is image information in the constructed low-rank, low-texture matrix, there are often two neighboring eigenvalues that are very different, which is called the eigenvalue jump. Usually, the eigenvalues above the jump point represent the image information. Therefore, we added a jump point judgment criterion to further constrain the choice of eigenvalues.

(18) $$\begin{array}{l} Eg(io)>5mean(Eg(2:iq-2))\\ Eg=\sum (2:iq)-\sum (1:iq-1) \end{array}$$

where *∑* represents the eigenvalue vector in descending order. Judging from the above equation, the number of eigenvalues satisfying the conditions is *io* and *io* ≤ *iq*. Then, the number of eigenvalues that satisfy the conditions is

(19) tk=min(ip,io).

Therefore, the standard deviation of noise is calculated as

(20) $$\sigma =\sqrt{\frac{1}{tk}\sum_{i=1}^{tk}\sigma_{i}^{2}}.$$

### 2.3. Proposed Algorithm

When estimating the noise level, it is necessary to first construct a low-rank sub-image block matrix, calculate the eigenvalues of this low-rank matrix, and then calculate the standard deviation of the noise according to the eigenvalues. In this calculation, there must be a certain number of pixels in the low-rank matrix. If the number of pixels is extremely small, the estimation results will be inaccurate and unreliable.

Suppose there are *n* independent and identically distributed variables *a_i_*, *I* = 1…*n*, and *a_i_* ~ *N*(0,*σ*^2^). Let $Z=\sqrt{\frac{a_{1}^{2}+a_{2}^{2}+\ldots +a_{n}^{2}}{n}}$, then $Z∼N(\sigma ,\frac{\sigma^{2}}{2n})$. According to the property of normal distribution, assuming that the error tolerance of noise estimation is FD, the minimum number of pixels required is

(21) $$n=\eta_{2}\frac{\sigma^{2}}{2dlt^{2}},$$

where *η*_2_ represents the scaling factor and *dlt* represents the error tolerance parameter.

First, the initial low-rank subblock *Y_l_* = {*y*_1_,*y*_2_… *y_l_*} ∈ *R^m^*^×*l*^ is obtained using the adaptive clustering method based on dichotomy merge, as shown in Algorithm 1. There may also be misclassified sub-images in the initial low-rank subblock. Therefore, it is necessary to further judge each image subblock vector in the initial low-rank subblock. We choose the image subblock with the smallest standard deviation as the reference subblock vector and calculate the Euclidean distance $d_{l}^{cj}$ between other image subblock vectors in the class and this reference subblock vector as the similarity measure index. When it is less than a certain threshold, the two subblock vectors are considered similar, and the calculation of this threshold is expressed as

(22) $$\tau =\eta_{1}\sqrt{2}\sigma ,$$

where *η*_1_ is a scaling factor. The similarity measure function between all sub-image vectors and the reference image is calculated, and all sub-image vectors satisfying $d_{l}^{cj}\leq \tau$ are retained to construct a new low-rank subblock *Y_k_* = [*y*_1_, *y*_2_… *y_k_*]^′^ ∈ *R^m^*^×*k*^. When the number of newly constructed low-rank subblocks m × k is less than *n*, the scaling factor *η*_1_ is enlarged to increase the number of columns *k* of the new low-rank subblocks.

Then, the dissimilar row vectors are screened out using Algorithm 2, and the new pixel-level low-rank subblock matrix $Y_{k}^{iq}$∈ *R^iq^*^×*k*^ is obtained by further optimizing the low-rank subblock matrix. Notably, when the noise is high, the number of similar row vectors calculated by Algorithm 2 may be small; therefore, we need to provide a minimum number of rows.

The pixel-level low-rank, low-texture sub-image matrix *Y_wp_* ∈ *R^iq^*^×*wp*^ is obtained using the low-texture selection method based on gradient covariance for the pixel-level low-rank subblock matrix.

Finally, the singular value selection method is used to select the singular value, and the standard deviation of the noise is calculated using Equation (23). The algorithm is summarized in Algorithm 3.
**Algorithm 3**: Our methodInput: original noise image *Y,* scaling factor *η,* scaling factor *η*_1_, scaling factor *η*_2_, minimum number of similar rows N, image block size *c,* maximum number of iterations *K,* error tolerance parameter *dlt,* confidence factor *δ.*Output: estimated standard deviation of noise.Steps:1. Calculate the initial standard deviation σ_0_ of noise using the algorithm in [38];2. Construct the initial low-rank sub-image matrix *Y_l_* = {*y*_1_,*y*_2_…*y_l_*} ∈ *R^m^*^×*l*^ using algorithm 1;3. Calculate the threshold $\tau =\eta_{1}\sqrt{2}\sigma_{0}$, select the sub-image with the minimum standard deviation as the reference sub-image vector, use the Euclidean distance as the criterion, retain the sub-image blocks whose Euclidean distance is less than the threshold in *Y_l_* as the new low-rank sub-image matrix *Y_k_* = [*y*_1_, *y*_2_… *y_k_*] ∈ *R^m^*^×*k*^, and if the least number of elements in the low-rank matrix is $n=\eta_{2}\frac{\sigma_{0}^{2}}{2dlt^{2}}$, run step 4, otherwise *η*_1_ = *η*_1_
*+* 0.01, and then rerun step 3;4. Use Algorithm 2 to construct pixel-level low-rank image subblocks $Y_{k}^{iq}$∈ *R^iq^*^×*k*^;5. For the *i*th image subblock, *I* = 1:*k*1.Calculate texture intensity $\xi_{i}=tr(C_{i})$;2.$\tau =\sigma_{0}^{2}Gamma^{-1}(\delta ,\frac{iq}{2},\frac{2}{iq}tr(FL_{h}^{\prime }FL_{h}+FL_{v}^{\prime }FL_{v}))$;3.When $\xi_{i}\leq \tau$, denote the *ith* image subblock as a low-texture sub-block;End for. Output the pixel-level low-rank low-texture sub-image matrix *Y_wp_* ∈ *R^iq^*^×*wp*^;6. If *iq* × *wp* < *n,* then go back to step 3;7. If *iq* × *wp* < *n* and all sub-blocks participate in the calculation, increase the texture intensity threshold and return to step 5;8. Perform SVD for *Y_wp_*;9. Through the judgment criteria, $if\{\begin{array}{cc} mean(\sum )\geq median(\sum ) & t=1\\ mean(\sum )<median(\sum ) & t=0 \end{array}$ and $\begin{array}{l} Eg(io)>5mean(Eg(2:iq-2))\\ Eg=\sum (2:iq)-\sum (1:iq-1) \end{array}$, obtain the number of eigenvalues, *ip* and *io*;10. Obtain tk=min(ip,io)
11. Calculate the standard deviation of the noise $\sigma =\sqrt{\frac{1}{tk}\sum_{i=1}^{tk}\sigma_{i}^{2}}$


## 3. Experiment

The experiments in this section have two main purposes: (1) to verify the effectiveness of the noise estimation algorithm proposed in this study, and (2) to verify the performance of the proposed noise estimation algorithm and the existing state-of-the-art noise estimation algorithm. To this end, the three most commonly used datasets in the field of image processing were selected: the TID2013 [46], Set12 [47], and BSD68 [48] datasets. In this study, we add additive white Gaussian noise with mean 0 and standard deviations σ = 10, 20, 30, 50, 70, 85, 100, 110, 120, 130, 140, 150, 160, 170, 180, 190, and 200. To verify the second purpose, we selected the four most commonly used and advanced noise estimation algorithms, namely, those based on statistics [38], weak texture area [34], pixel-level matrix [36], and WNNM [49], for comparison with the proposed algorithm. The original codes for the four algorithms can be downloaded from the author’s website. The dataset and the code can be found online at http://ponomarenko.info/tid2013.htm (accessed on 13 November 2022), https://www2.eecs.berkeley.edu/Research/Projects/CS/vision/bsds (accessed on 16 November 2022), https://github.com/njusthyk1972/NLH (accessed on 13 November 2022), https://www.mathworks.com/matlabcentral/fileexchange/64519-natural-image-noise-level-estimation-based-on-flat-patches-and-local-statistics (accessed on 13 November 2022), and http://www4.comp.polyu.edu.hk/~cslzhang/code/MCWNNM.zip (accessed on 13 November 2022). To be fair, we directly used the optimal parameters debugged by the author and used the same version of the MATLAB software and the same computer (16 GB RAM, 3.6 GHZ CPU, and an Inter (R) Core (TM) i3-8100 processor). Our algorithm was also written in MATLAB.

To evaluate the estimation results of the noise standard deviation, it is necessary to carry out a quantitative index evaluation. We selected the three most commonly used evaluation indicators in the field of noise estimation: bias (bias), standard deviation (STD), and mean square error (MSE), which reflect the accuracy, robustness, and overall performance of the algorithm, respectively. The calculation formulas are as follows:

(23) $$\begin{array}{l} \mathrm{MSE}=\sqrt{{\mathrm{STD}}^{2}+Bias^{2}}\\ \mathrm{Bias}=E\left|\sigma -E(\hat{\sigma })\right|\\ \mathrm{STD}=\sqrt{E{\left[\hat{\sigma }-E(\hat{\sigma })\right]}^{2}} \end{array}$$

Note that smaller bias, STD, and MSE values indicate better performance. These parameters must be determined before starting the simulation. The initial parameters of the proposed algorithm are listed in Table 1. We set the maximum number of iterations K to 50.

**Figure 3:**
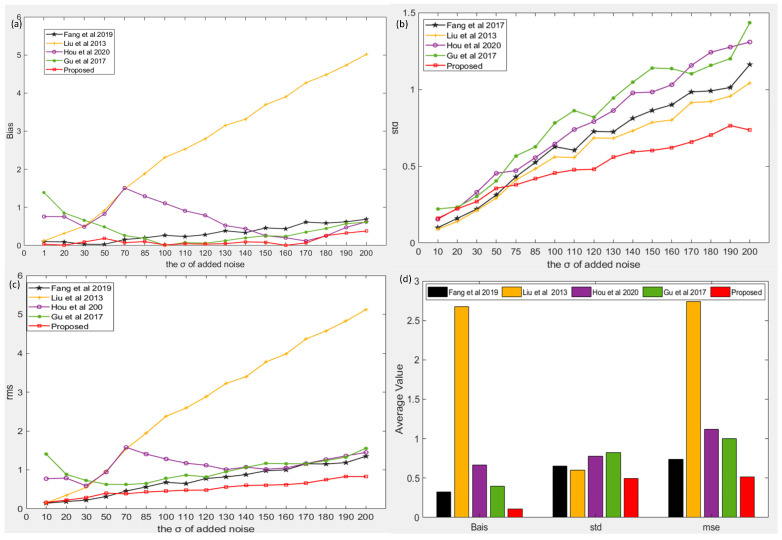
Comparison of the results of our algorithm and four existing algorithms [34,36,38,49] under Set12 dataset. (**a**) the bias of noise estimation results of five algorithm under different noise levels, (**b**) the std of noise estimation results of five algorithm under different noise levels, (**c**) the mse of noise estimation results of five algorithm under different noise levels, (**d**) the average value of noise estimation results of five algorithm under different noise levels.

The images in the Set12 dataset contain more or less homogeneous regions. Comparing the noise estimation results on the Set12 dataset, our algorithm is almost optimal for the three indexes of bias, STD, and MSE. As shown in Figure 3a, in terms of the bias index, except when the noise standard deviation is 30 and 50, the accuracy of the algorithm of [38] is slightly better than that of our proposed algorithm, which is the most accurate in other cases. As the standard deviation of the noise increases, the bias estimated by our algorithm is generally stable, whereas the bias estimated by the algorithm of [38] gradually increases. Although the bias estimated by the algorithm of [49] is close to that of our algorithm in some cases of high noise, it is larger in the case of low noise. Therefore, the accuracy of our algorithm is the best. As shown in Figure 3d, the bias estimated by our algorithm decreased by 3–24 times compared to the other algorithms. The bias estimated by our algorithm was 0.1093, while the bias estimated by other algorithms was 0.3988, 0.6668, 2.673, and 0.3254, respectively. In terms of the STD index, the algorithms of [38] and [34] are slightly more robust than our proposed algorithm only when the noise standard deviation is less than 50. In addition, our algorithm has obvious advantages over other algorithms, which means that the robustness of our algorithm is better than that of other algorithms. Owing to the advantages of our algorithm in these two indexes, it also has obvious advantages in the MSE index. From Figure 3d, STD and MSE were reduced by approximately 1.2–1.6 and 1.4–5.3 times, respectively. The STD estimated by our algorithm was 0.4971, while the STD estimated by other algorithms was 0.8219, 0.7764, 0.6035, and 0.6561, respectively. The MSE estimated by our algorithm was 0.5155, while the MSE estimated by other algorithms was 1.002, 1.12, 2.743, and 0.7366, respectively.

**Figure 4:**
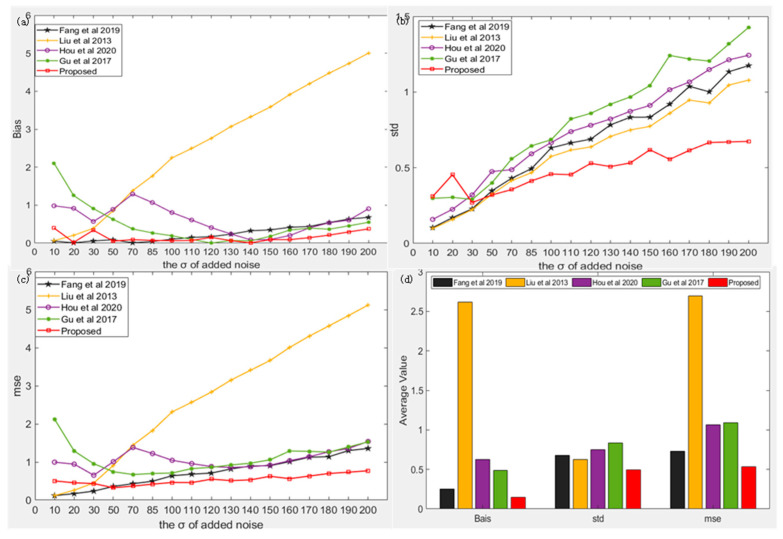
Comparison of the results of our algorithm and four existing algorithms [34,36,38,49] under TID2013 dataset. (**a**) the bias of noise estimation results of five algorithm under different noise levels, (**b**) the std of noise estimation results of five algorithm under different noise levels, (**c**) the mse of noise estimation results of five algorithm under different noise levels, (**d**) the average value of noise estimation results of five algorithm under different noise levels.

**Figure 5:**
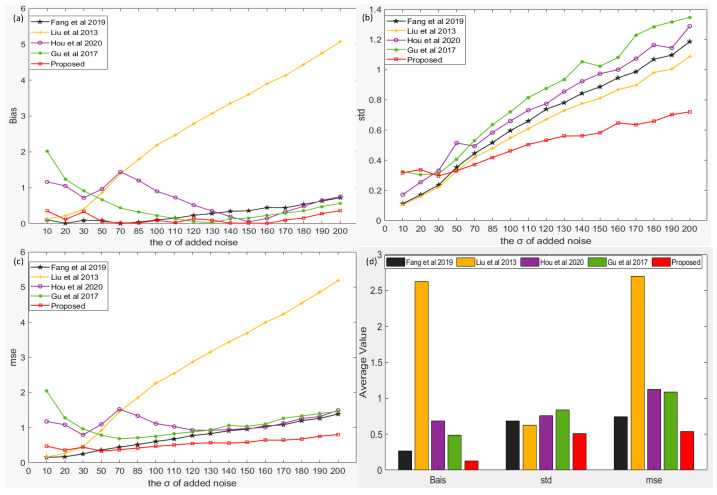
Comparison of the results of our algorithm and four existing algorithms [34,36,38,49] under BSD68 dataset. (**a**) the bias of noise estimation results of five algorithm under different noise levels, (**b**) the std of noise estimation results of five algorithm under different noise levels, (**c**) the mse of noise estimation results of five algorithm under different noise levels, (**d**) the average value of noise estimation results of five algorithm under different noise levels.

The image complexity of the TID2013 dataset is higher than that of the Set12 dataset, and some images have no homogenization regions. The calculation results in Figure 4c show that our algorithm still has clear advantages. When the standard deviation of the noise is less than 50, the overall performance of our algorithm is slightly worse than that of the algorithms of [34,38]. However, when the standard deviation of noise is greater than 50, the advantages of our algorithm begin to be detected gradually, and the advantages become more evident with the increase in noise. As can be observed from Figure 5d, overall, the MSE of our algorithm is reduced by 1.3–5 times. The MSE estimated by our algorithm was 0.532, while the MSE estimated by other algorithms was 1.093, 1.063, 2.694, and 0.7289, respectively. As shown in Figure 4b, similar to the performance in the Set12 dataset, the robustness advantage of our algorithm is gradually reflected once the standard deviation of noise is greater than 50, and the advantage gradually increases with an increase in noise. In terms of the bias index, overall, our algorithm is inferior to the algorithm of [38] when the noise standard deviation is less than 85; however, with the increase in noise, our algorithm gradually performs better than that algorithm. When the standard deviation of the noise is greater than 85, our algorithm is always optimal, except the algorithm of [49] outperforms our algorithm when the noise standard deviation is 120. Figure 4d shows that the deviation and standard deviation of our algorithm were reduced by approximately 1.7–17.4 and 1.2–1.7 times, respectively. The bias estimated by our algorithm was 0.1501, while the bias estimated by other algorithms was 0.4858, 0.625, 2.616, and 0.2538, respectively. The STD estimated by our algorithm was 0.4943, while the STD estimated by other algorithms was 0.8355, 0.7492, 0.6238, and 0.6752, respectively.

The images in the BSD68 dataset are closer to reality and are more complex; therefore, we further tested the performance of our algorithm on this dataset. As can be observed from Figure 5a, when the standard deviation of noise is less than 50, our algorithm is only slightly worse than the algorithm of [38] but better than the other algorithms. However, as the standard deviation of noise increases, our algorithm gradually becomes better than the algorithm of [38], and the advantage gradually increases with an increase in noise. When the standard deviation of noise is 10, the algorithm of [34] is better than our algorithm, but with the increase in noise, our algorithm outperforms the algorithm of [34] significantly. When the standard deviations of noise are 120 and 130, the algorithm of [49] is slightly better than our algorithm, and our algorithm has obvious advantages in other cases. In terms of the STD and MSE indices, similar to the performance on other datasets, when the standard deviation of noise is greater than 50, our algorithm is more robust than other algorithms with better overall performance, and the advantage gradually increases with increasing noise. As shown in Figure 5d, the overall deviation, standard deviation, and root mean square of our algorithm were reduced by 2.1–20.4, 1.2–1.6, and 1.4–5 times, respectively. The bias (STD/MSE) estimated by our algorithm was 0.1286 (0.508/0.5393), while the bias (STD/MSE) estimated by other algorithms was 0.4852 (0.8349/1.089), 0.6851 (0.7609/1.125), 2.621 (0.6288/2.7), and 0.2698 (0.6839/0.7434), respectively.

It can be concluded from Figure 3, Figure 4, and Figure 5 that, on these three datasets, the performance of our proposed algorithm was highly stable, and the difference in each index indicates that our algorithm can be applied in a variety of situations, rather than being limited to a certain scenario. It can be seen from Table 2 that our algorithm outperforms the other four algorithms. The bias, STD, and MSE estimated by our method were reduced by 2.18, 1.23, and 1.39 times, respectively. However, due to the clustering operation of our algorithm, the running time of our algorithm was much longer than that of other comparison algorithms. The average running time of the other four algorithms was less than 1 s (the average running times of methods [34,36,38,49] were 0.12 s, 0.28 s, 0.68 s, and 0.03 s, respectively), while the average running time of our algorithm was 15.37 s.

## 4. Conclusions

In this study, we proposed a novel noise estimation algorithm based on pixel-level low-rank, low-texture sub-image block construction and noise eigenvalue averaging. First, we used the adaptive clustering method based on dichotomy merge to construct a low-rank sub-image matrix. Then, a pixel-level low-rank, low-texture sub-image block matrix was constructed using the construction method of the pixel-level low-rank sub-image block matrix and the selection method of the low-texture sub-image block based on gradient covariance, and the calculation method for the minimum number of pixels to be included in the matrix was presented. Finally, based on the low-rank property of the matrix, the eigenvalues of the noise were selected using the matrix eigenvalue selection method, and the standard deviation of the noise was then calculated using the mean method. The experimental results show that the noise standard deviation obtained in this manner has higher accuracy and robustness.

To further demonstrate the performance of the proposed algorithm, it was compared with four existing state-of-the-art algorithms on multiple datasets and a wide range of noise levels. Our algorithm achieved the highest accuracy and robustness in most cases. The bias, STD, and MSE estimated by our method were reduced by 2.18, 1.23, and 1.39 times, respectively. Specifically, for higher noise levels, the advantages of our algorithm were more obvious. Moreover, the performance of our algorithm on these different datasets was highly stable. This indicates that our algorithm is relatively independent of the scenario and has wider applicability.

## Figures and Tables

**Table 1:** Initial parameters of our algorithm.

*σ*	*c*	*δ*	*dlt*	*η*	*η*_1_	*η*_2_	*N*
≤15	10	1-10^−6^	0.02	0.93	3	1.2	70%
≤35	10	1-10^−6^	0.02	0.93	3	1.2	70%
≤90	10	1-10^−6^	0.1	0.99	3	1.2	70%
≤120	10	1-10^−6^	0.1	0.99	3	1.2	70%
≤150	10	1-10^−6^	0.1	1	3	1.2	70%
≤200	10	1-10^−6^	0.1	1.01	3	1.2	70%

**Table 2:** Results of the compared methods for all datasets.

Method	Bias	STD	MSE
Method [38]	0.2829	0.6717	0.7363
Method [36]	0.659	0.7622	1.103
Method [34]	2.637	0.6187	2.712
Method [49]	0.4566	0.8308	1.061
Our Method	0.1293	0.4998	0.5289

## Data Availability

The datasets used in this paper are publicly available [46,47,48] or can be obtained on the website: http://ponomarenko.info/tid2013.htm (accessed on 13 November 2022), https://www2.eecs.berkeley.edu/Research/Projects/CS/vision/bsds (accessed on 13 November 2022).
